# Treatment of Acute Compartment Syndrome Sequela of the Leg: A Case Report Demonstrating Negative Pressure Wound Therapy with Instillation and Dwell Utilizing a Novel Dressing and Serial Automated Suction Blister Epidermal Harvesting and Grafting

**DOI:** 10.7759/cureus.3443

**Published:** 2018-10-12

**Authors:** Ralph J Napolitano

**Affiliations:** 1 Podiatry and Wound Care, OrthoNeuro, Columbus, USA

**Keywords:** wound care, compartment syndrome, orthopedic trauma, general surgery, skin grafting, emergency medicine, cellutome, negative pressure wound therapy with instillation and dwell, negative pressure wound therapy, cleanse choice™ dressing

## Abstract

Compartment syndrome is a pathologic condition in which a closed anatomical compartment’s pressure, most often in the arms and legs, increases to such an extent that the microcirculation of the tissues in that compartment is diminished either acutely or subacutely over time. Such vascular compromise, if untreated, may result in tissue necrosis and muscle and nerve damage. If limb tissue damage is severe enough, amputation of the involved extremity may be necessary. Symptoms of compartment syndrome may include severe pain, diminished or non-palpable pulses, paralysis of the involved extremity, and dermatologic sequela. There are two types of compartment syndrome: acute and chronic. The former is considered a surgical emergency in most cases requiring prompt diagnosis and treatment in order to release intracompartmental pressure thus decreasing the chance of significant tissue damage. In this article, the author presents a novel case in which acute compartment syndrome sequela of the lower leg was successfully treated with surgery, negative pressure wound therapy with instillation and dwell utilizing a novel dressing and serial automated suction blister epidermal harvesting and grafting. The outcome was favorable, resulting in complete limb preservation and return to normal function.

## Introduction

Compartment syndrome history

Medical descriptions of compartment syndrome have been well documented in the literature for more than 100 years. Volkmann, in 1881, was the first to describe the clinical findings of muscle death and contracture due to prolonged muscle ischemia in his paper Die ischaemischen Muskellähmungen und Kontrakturen (Article in German) [1]. He thought that some kind of external compression force, specifically splints, led to arterial inflow obstruction, which caused myonecrosis. Using a canine model more than four decades later, Jepson showed that external compression forces, specifically constricting bandages, could lead to Volkmann's limb deformities; however, if surgery was performed to drain the collected pathologic serosanguineous fluids, the muscle tissue was spared and the dogs could walk more normally [2]. In 1940, Griffiths suggested another specific idea regarding limb contracture from compartment syndrome other than external compression [3]. He described several cases in which brachial artery embolectomies were necessary and in whom Volkmann's contractures developed. He suggested that the cause of compartment syndrome was arterial insufficiency. He also described clinical criteria for the diagnosis of compartment syndrome: pain with passive movement, painful onset, pallor, puffiness, and eventual pulselessness. Over the years, these signs and symptoms have been refined and are known as the “Five Ps” that are often associated with compartment syndrome: pain, pallor paresthesia, pulselessness, and paralysis. In the 1970s, investigators began to focus on the basic science, clinical course, and treatment of compartment syndrome. Rorabeck and Macnab recognized that either arterial insufficiency or venous obstruction could lead to compartment syndrome [4]. Utilizing radioactive reagents, they showed that the clearance of radioactive technetium from an anatomical compartment was inversely related to the compartment pressures. In turn, these data were directly related to blood flow. They demonstrated that blood flow to a compartment was quickly restored following fasciotomy and fluid release. They also observed a reperfusion injury after decompression. Whitesides et al. noted the importance of timeframe with respect to ischemia [5]. Using electron microscopy to analyze canine muscle, they observed that less than 5% of muscle cells were damaged after four hours of ischemia; however, nearly 100% of muscle cells were damaged after eight hours of ischemia. Matsen et al. showed that compartment syndrome caused nerve dysfunction over time [6]. As nerve conduction velocity steadily diminished under anatomical compartment pressure, paresthesia and hypoesthesia were observed initially. Signs and symptoms escalated over time if pressures were not released, resulting in motor weakness and anesthesia. They also described that excessive elevation of a severely injured extremity might increase the risk of compartment syndrome. They described the connection between elevation and diminished arteriolar pressure resulting in tissue hypoxia. More recently, Heppenstall et al. have carried out a number of significant experiments focusing on diagnosing compartment syndrome more accurately by defining the relationship between the injury time and compartment syndrome pathology [7].

Pathophysiology and treatment of extremity compartment syndrome 

Compartment syndrome develops from either intracompartmental swelling or external compression. Both of these processes lead to elevated tissue pressures. As pressure increases, local blood flow decreases. The pathophysiology of compartment syndrome is directly related to the increased fluid pressure in a closed anatomical compartment, most often in the arms and legs. Pressure increases to such an extent that the microcirculation of the tissues in that compartment diminishes either acutely or subacutely over time [8]. Such vascular compromise, if untreated, may result in tissue necrosis, muscle and nerve damage. If tissue damage is severe enough, and an extremity is involved, amputation of the involved extremity may be necessary. It should be noted that the classic "Five Ps" of compartment syndrome (pain, pallor paresthesia, pulselessness, and paralysis) may present in their entirety in more severe cases, or partially in varying degrees. There are two types of compartment syndrome: acute and chronic. The former is considered an emergency requiring prompt diagnosis and treatment in order to decrease or surgically release intracompartmental pressure; thus, decreasing the chance of significant tissue damage. Chronic, also known as exertional, compartment syndrome is thought to be a result of hyperemia in the setting of exercise that results in muscular swelling in a closed anatomical compartment. The exact cause of chronic compartment syndrome is not completely understood, but symptomatology is markedly less severe compared to acute compartment syndrome. Symptoms can be reversed with rest and discontinuing the exercise. Prophylactic fasciotomy may be indicated if symptomatology persists but does carry certain risks such as wound closure complications. Initial management of acute compartment syndrome involves removing any casts or dressings on the affected limb. Close monitoring of the patient and the limb is necessary. The limb should not be elevated but should rather be kept at heart level to restore normal perfusion to the tissues. If symptomatology does not improve, fasciotomy is indicated as an emergency procedure to decompress the compartments and prevent irreversible ischemic damage to muscles and other tissues. Mubarak and Hargens have suggested intracompartmental pressure (ICP) thresholds when considering fasciotomy. They have proposed that surgical fasciotomy is indicated in normotensive patients with ICP higher than 30 mmHg and in hypotensive patients with ICPs greater than 20 mmHg [9]. When the fasciotomy is performed, an incision large enough to adequately decompress all the compartments involved is necessary. Surgical decompression is not always indicated if compartment syndrome has been present for more than 48 hours and adequate function exists [10]. 

Negative pressure wound therapy with instillation and dwell time

Negative pressure wound therapy (NPWT) is a popular, well-documented treatment for the management of both acute and chronic wounds. An NPWT system promotes wound healing by drawing wound edges together, removing infectious materials and exudate, and it actively promotes the formation of granulation tissue. Its use in the medical community worldwide is diverse. Treatment of decubitus wounds, diabetic foot ulcers, venous leg wounds, and surgical dehiscence wounds with NPWT have all been well documented in the literature. This modality was first promoted in 1989 by Chariker et al. who described a suction drainage system for the treatment of incisional and cutaneous fistulae [11]. The system they described was different compared to modern NPWT systems. Their device used a gauze-filled dressing connected to a wall suction. Therapy pressure was set at 60-80 mmHg. They believed that their system was effective in removing excess wound fluid, potentiating the formation of granulation tissue, and reducing periwound skin damage. In the early 1990s, polyurethane sponges were favored for their positive effect on granulation tissue. Most of the devices currently in the market contain a similar open pore polyurethane dressing. Argenta and Morykwas pioneered the use of open pore polyurethane dressings coupled with subatmospheric pressure at 125 mmHg below ambient pressure [12]. This configuration forms the basis of today's contemporary NPWT device. Orgill and Bayer described four primary effects of NPWT on wound healing: macro-deformation and wound contracture, stabilization of the wound environment, edema reduction and exudate removal, and micro-deformation leading to cellular proliferation on the wound surface [13]. It is well known that all chronic wounds contain bacteria, but wound healing can occur even in the presence of bacteria. In fact, certain bacteria, such as Staphylococcus aureus, appear to aid wound healing [14]. Therefore, it seems that not their mere presence but the type and number of microorganisms determine their influence on wound healing. Addressing this bacterial bioburden can be accomplished by a variety of methods ranging from systemic antibiotic treatment, serial debridements, and using wound dressings impregnated with antimicrobial reagents. Negative pressure wound therapy with instillation and dwell (NPWTi-d) combines the benefits of negative pressure wound therapy with automated topical wound solution instillation and removal. Although this particular modality is not a therapy for the treatment of wound infection or a mechanism to deliver intravenous antibiotics, it can facilitate wound care in other ways. Such benefits include cleansing the wound through the instillation of topical wound cleansers that can help soften and loosen wound debris and delivering topical antiseptic/antimicrobial wound solutions that can help manage bacterial bioburden population [15]. Both NPWT and NPWTi-d have been utilized in the treatment of wound-associated compartment syndrome surgery [16].

Automated suction blister epidermal harvesting and grafting

Epidermal grafting has several advantages over full-thickness or split-thickness skin grafts in the treatment of complex non-healing wounds in some cases. These include essentially no donor site morbidity, minimal patient discomfort, and the ability to harvest tissue without surgery or anesthesia. Suction blister epidermal harvesting and grafting is a simple and effective skin grafting technique in which the epidermis is procured in an autologous fashion under suction and transferred to a recipient site. This work was first pioneered by Kiistala and Mustakallio in 1964 [17]. Since then, the technique has evolved over time and has been used to treat a variety of skin conditions and wounds. These include vitiligo, diabetic foot wounds, and pressure ulcers. For several years, the CelluTome™ Epidermal Harvesting System (KCI, an Acelity company from San Antonio, TX, USA) has been available for commercial use. This automated system combines heat and suction to produce epidermal blisters that can be harvested and transferred to a recipient site [18].

## Case presentation

**Figure 1:**
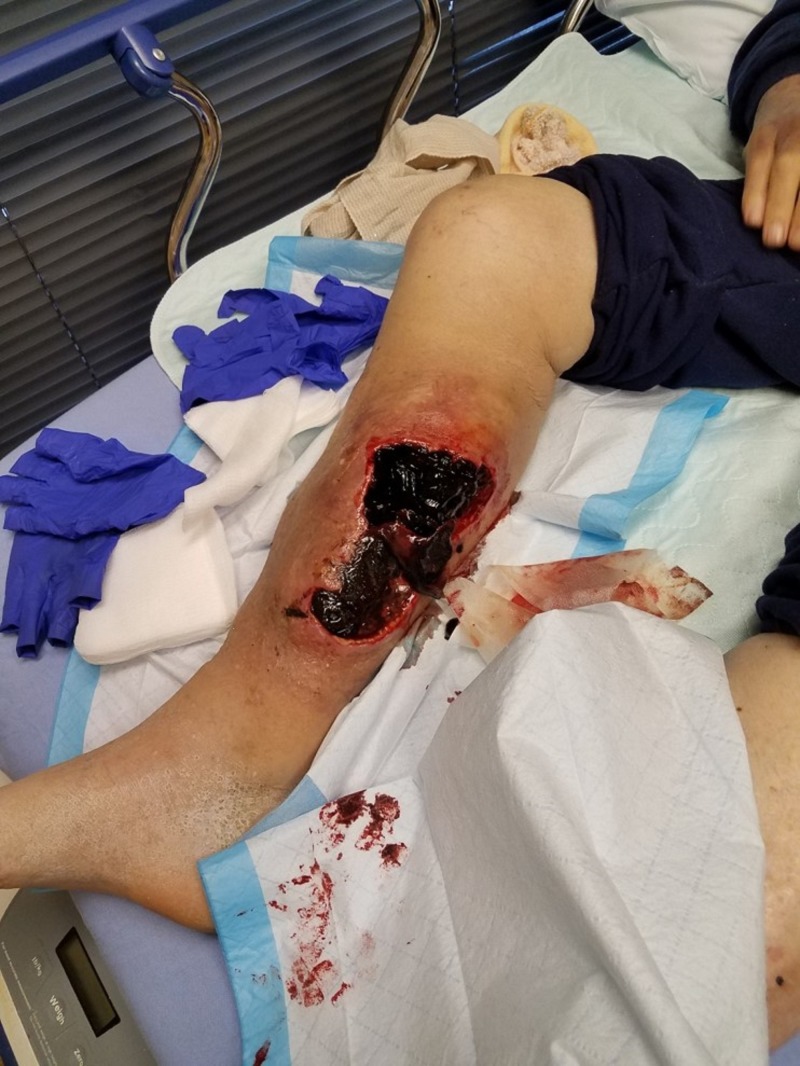
Initial presentation Medial lower leg with extensive soft tissue necrosis.

**Figure 2:**
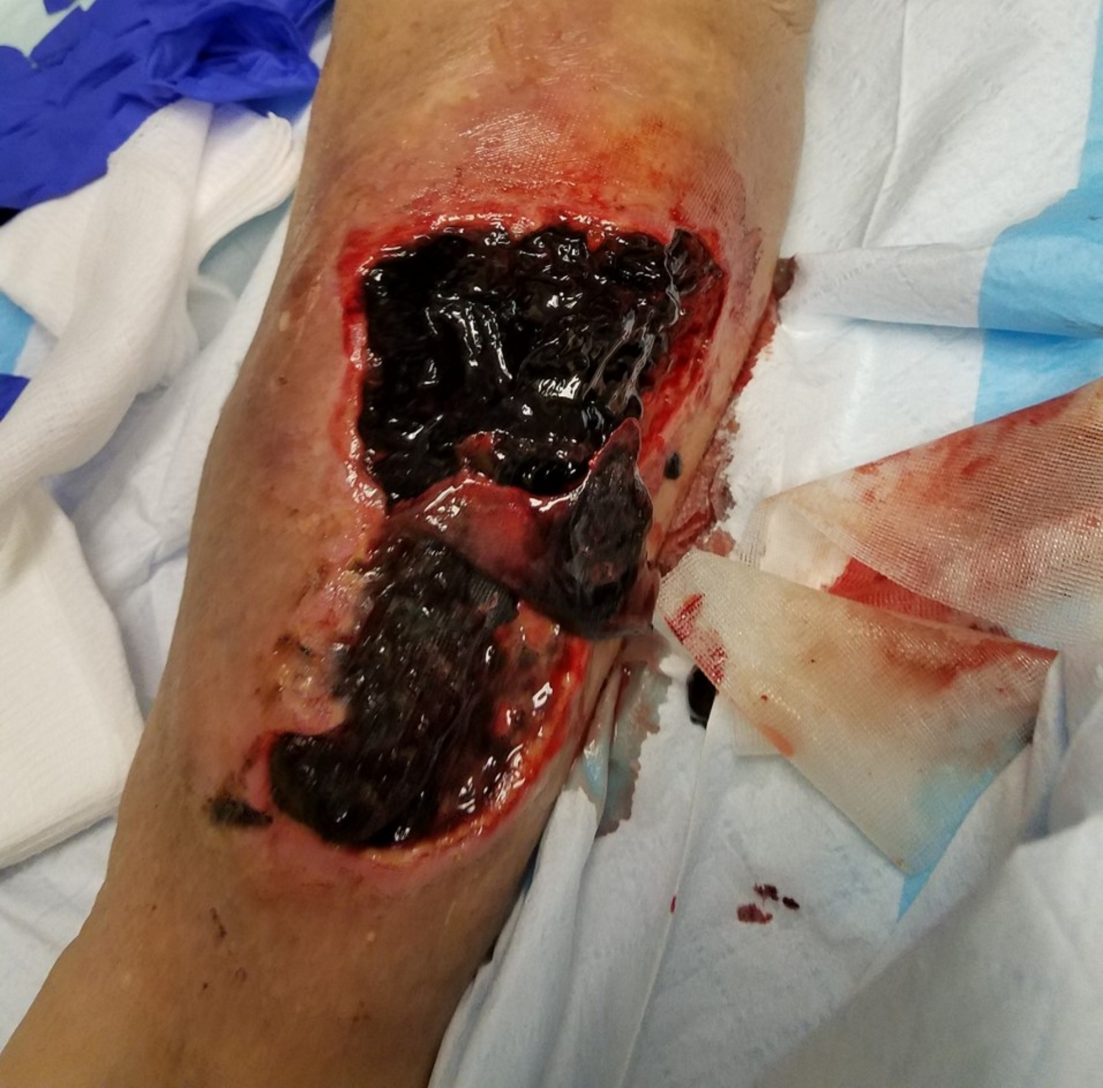
Initial presentation Close-up photograph showing extensive soft tissue necrosis.

An 83-year-old male, living alone and independently, presented to St. Ann’s Hospital Wound Clinic on December 29, 2017. Less than a week prior, he stated to the family that "I fell into a bar stool at home." Upon further exploration, the family felt that he fell asleep in his power recliner resulting in his right leg being lodged in the hinge portion of the footrest. Initial presentation as related by the patient and family included a hematoma on the medial portion of the right calf, tingling in the right leg and foot, and some weakness of the involved extremity. Watchful waiting was employed by the patient and family. Over the next 24-48 hours, the patient and family reported that the hematoma increased in size and severity resulting in significant wound involvement and tissue necrosis (Figures 1-2). This increase in the severity of symptoms prompted their visit to St. Ann’s Hospital Wound Clinic, Westerville, Ohio.

**Figure 3:**
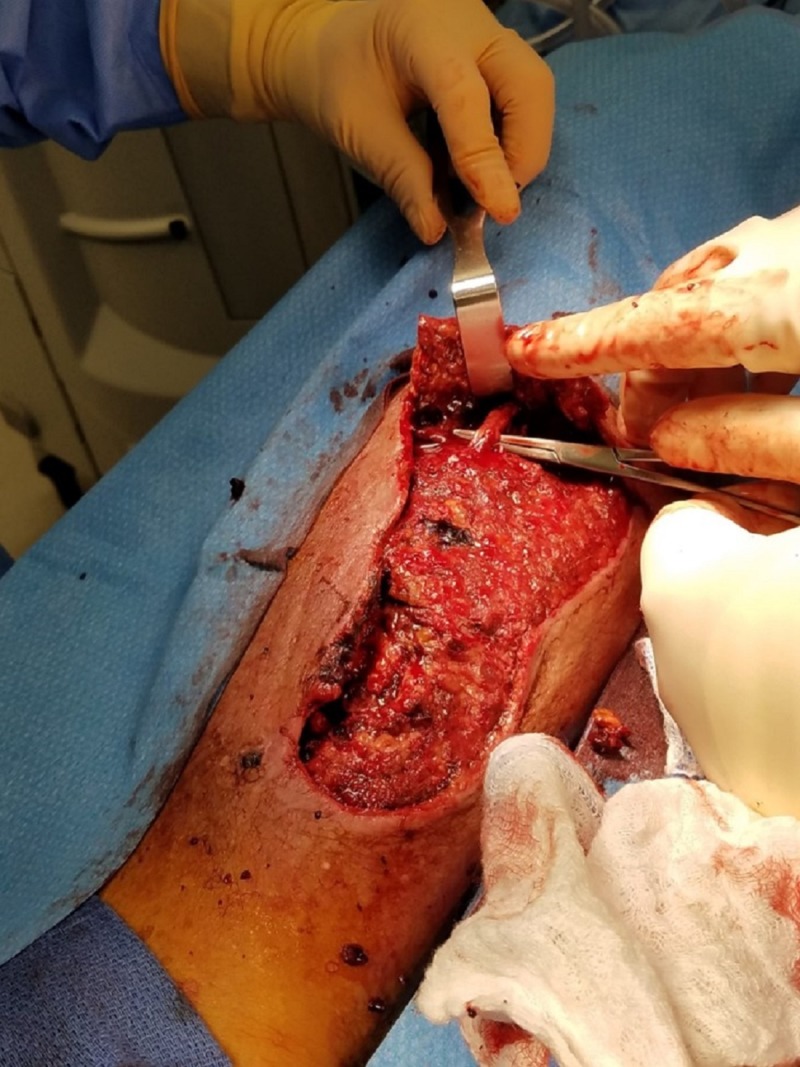
Proximal surgical site Surgical debridement demonstrating tibial nerve branch inspection and decompression.

**Figure 4:**
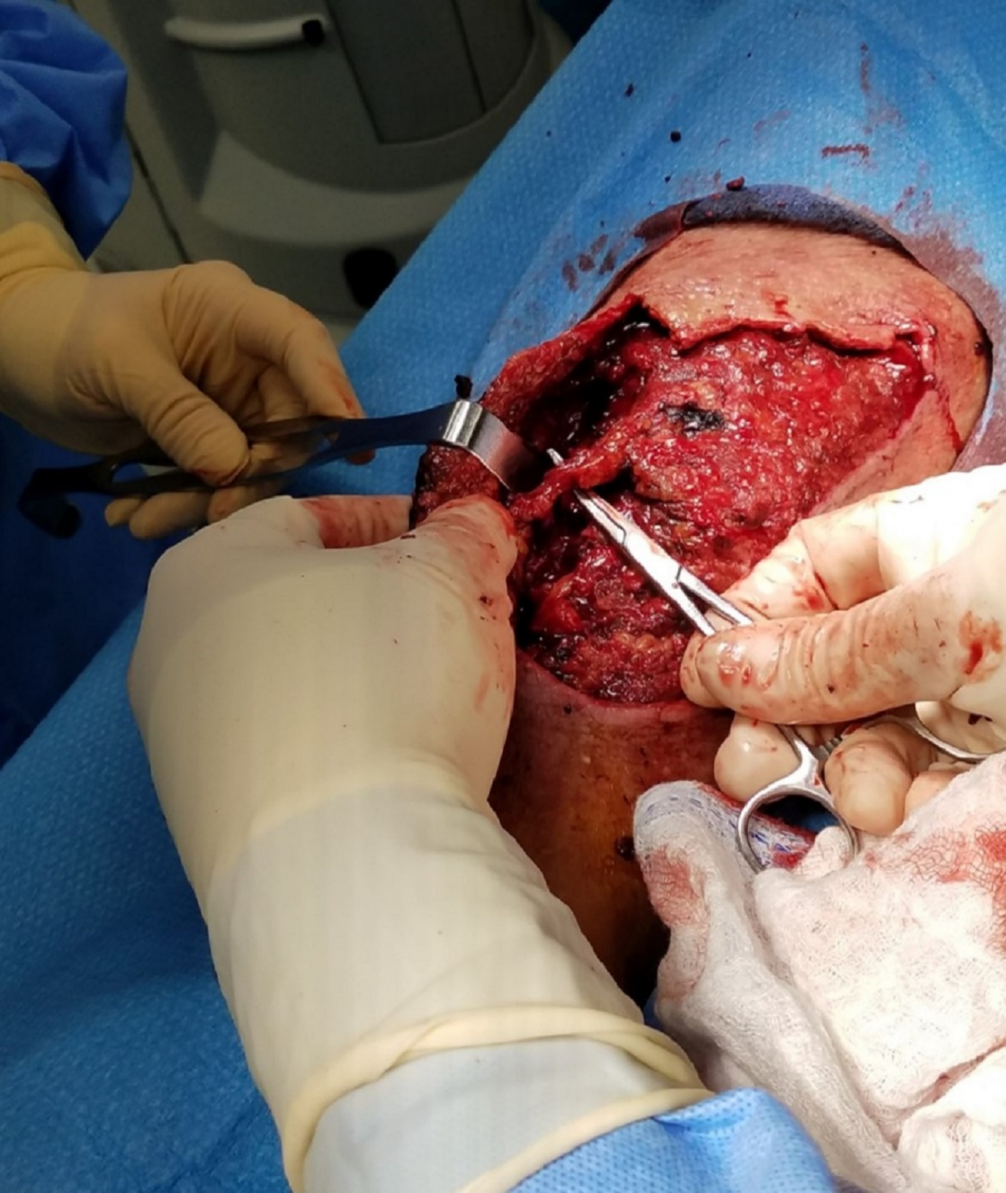
Distal surgical site Further debridement and mobilization of neurovascular structures.

The initial exam demonstrated extensive soft tissue damage with muscle necrosis and old gelatinous hematoma involvement. The neurologic exam demonstrated intact deep tendon reflexes, no sensation loss, but some mild subjective parasthesias. Vascular and orthopedic exams showed no gross deformity, an adequate range of motion with some guarding, and intact peripheral pulses with spongy edema. The patient was promptly admitted for appropriate medical workup in preparation for surgical debridement. Imaging failed to reveal any fractures and the full-body exam was without remark. Surgery included evacuation of the residual hematoma, extensive surgical debridement of necrotic tissues including muscle, mechanical cleansing with pulsed lavage, and deep tissue cultures (Figures 3-4).

**Figure 5:**
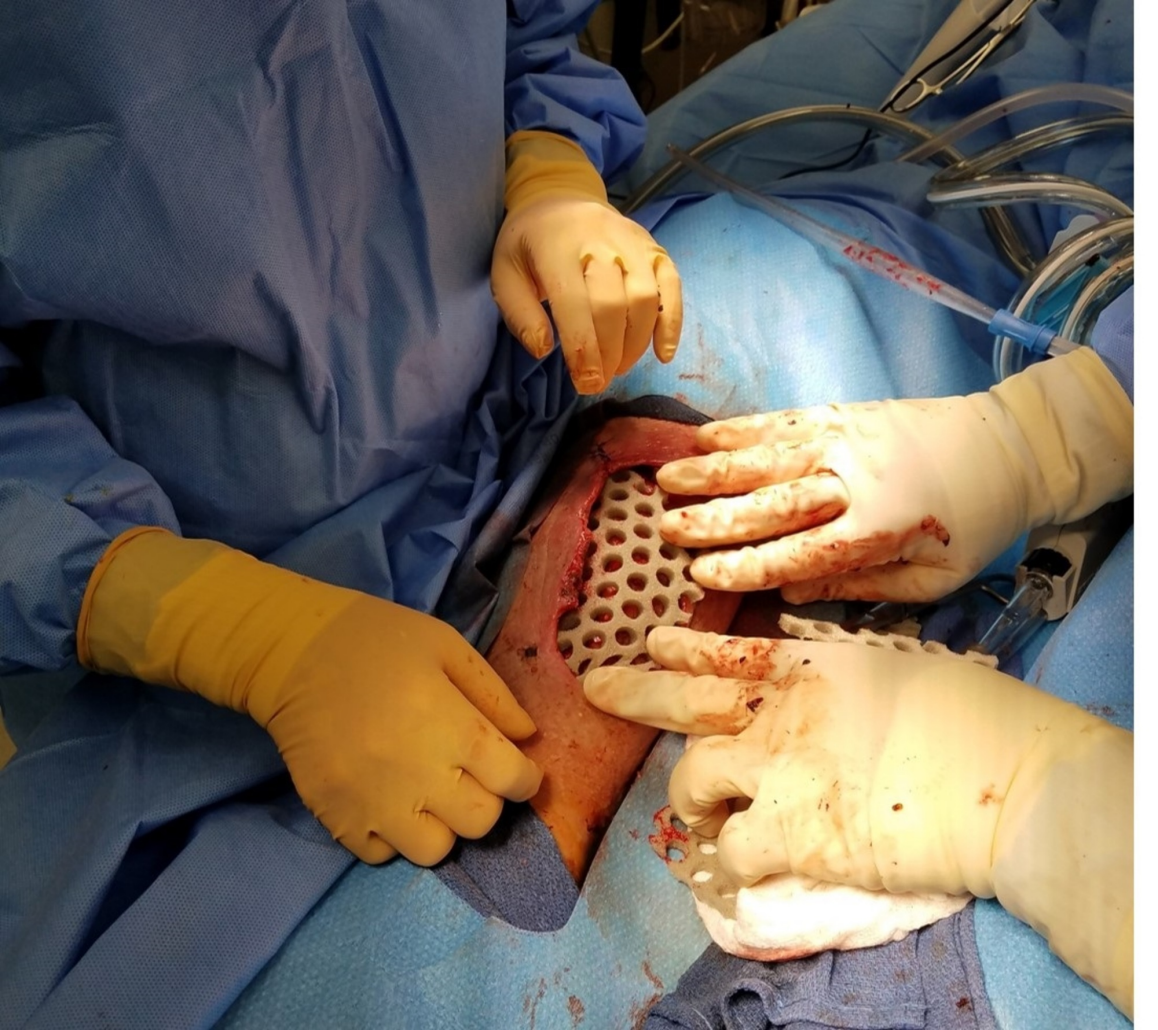
Primary pressing layer Placement of polyurethane reticulated open cell foam dressing with through holes (V.A.C. VeraFlo Cleanse Choice Dressing^TM^, KCI, an Acelity company, San Antonio, TX, USA).

**Figure 6:**
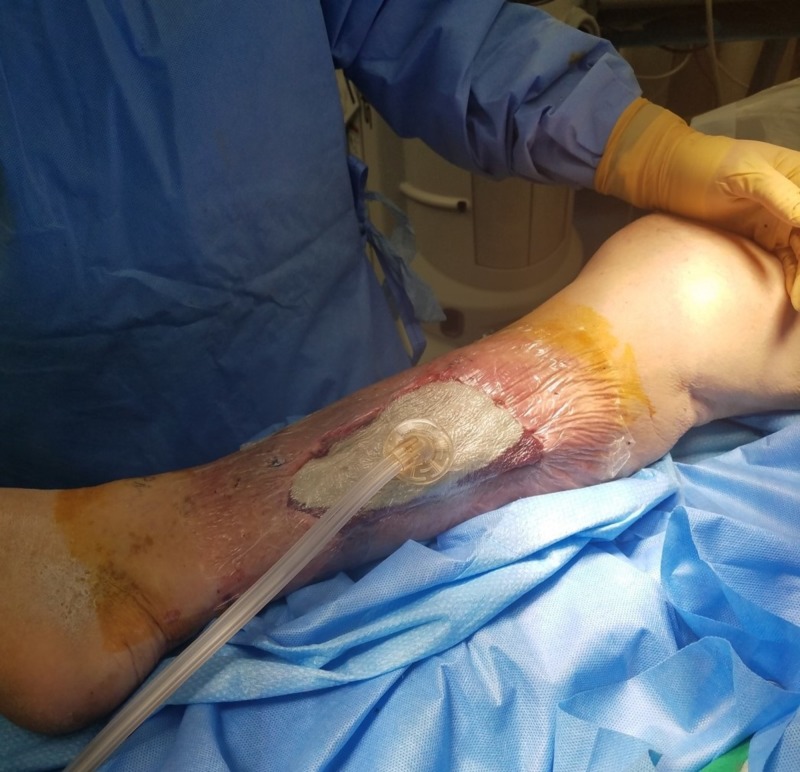
Complete negative pressure wound therapy device after draping and trackpad placement Negative pressure wound therapy with instillation and dwell initiated.

Once the surgery was completed, NPWTi-d was initiated in the operating room. The primary foam dressing layer was a novel dressing consisting polyurethane reticulated open cell foam with through holes (V.A.C. VeraFlo Cleanse Choice™ Dressing, KCI, an Acelity company, San Antonio, TX, USA). The second layer of thin polyurethane foam was placed over the through hole layer. Standard negative pressure wound therapy draping and trackpad placement were carried out (Figures 5-6).

**Figure 7:**
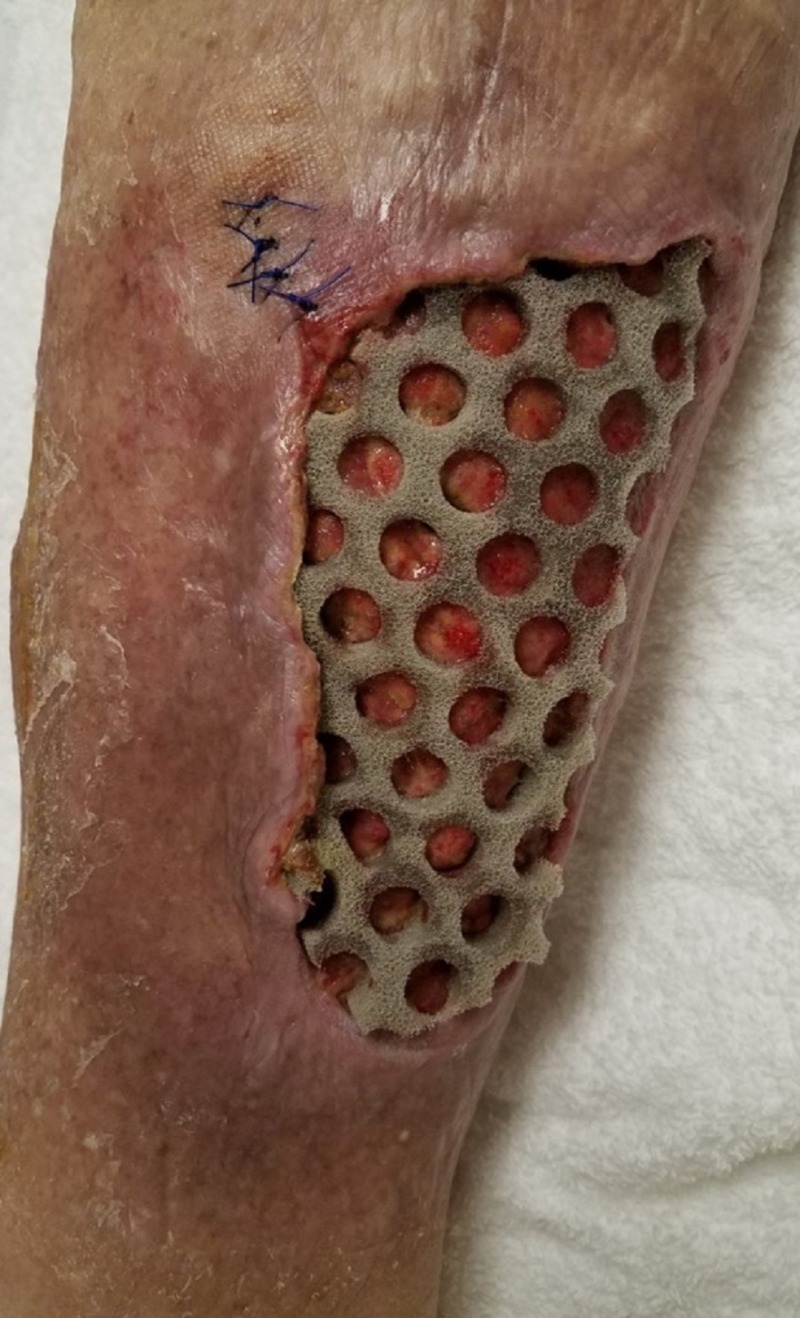
Surgical site at first dressing change Primary dressing with through holes in place before first complete takedown.

**Figure 8:**
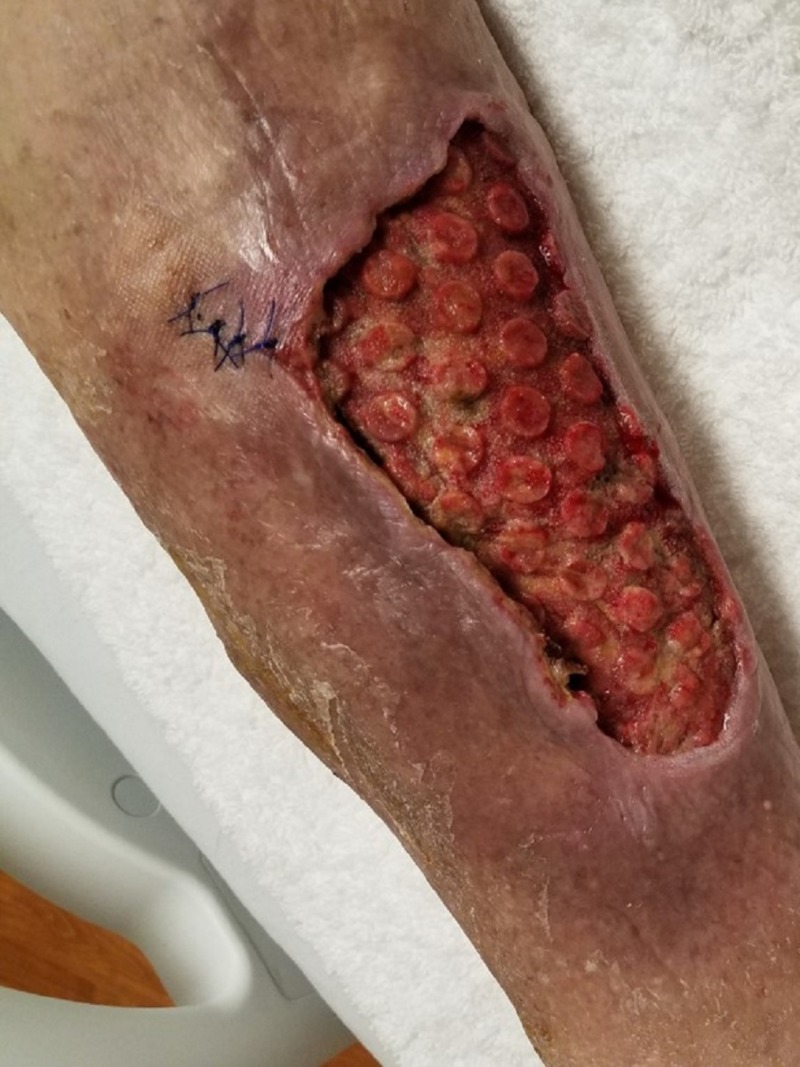
First postoperative dressing change Wound bed after 48 hours of negative pressure wound therapy with instillation and dwell time demonstrating significant hypergranular changes.

NPWTi-d was initiated by instilling normal saline that dwelled in the wound bed for eight minutes, followed by three-and-a-half hours of continuous -125 mmHg negative pressure. The amount of solution instilled was 30 ml. The patient received this therapy during his hospital stay of six days. An interval dressing change was performed at 48 hours postoperative. After 48 hours of therapy, significant hypergranular tissue plug changes were seen in the wound bed (Figures 7-8).

**Figure 9:**
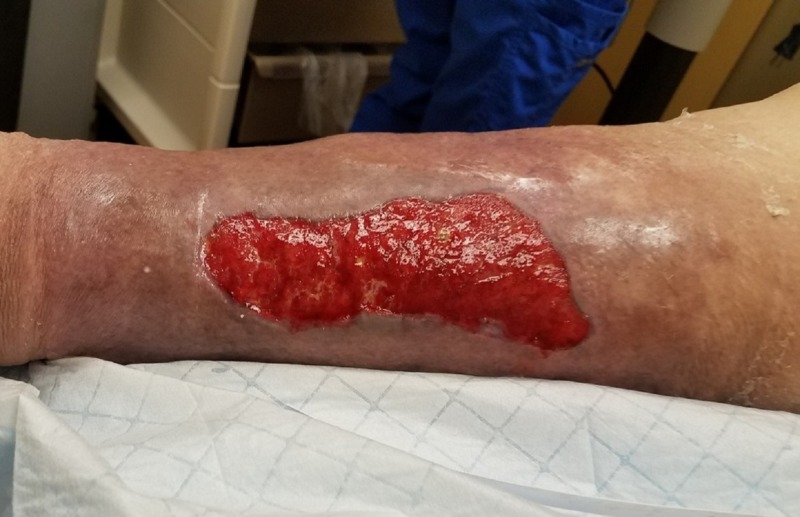
Wound after 21 days of negative pressure wound therapy (NPWT) Wound after three weeks of NPWT preceeded by six days of negative pressure wound therapy with instillation and dwell.

Surgical wound cultures were negative other than skin flora. Initial parenteral antibiotics were changed to oral antibiotics before hospital discharge. After six days of NPWTi-d, the patient was transferred to a skilled nursing facility and he continued to receive traditional NPWT without instillation (V.A.C. Freedom®, KCI, an Acelity company, San Antonio, TX, USA). The pressure was maintained at continuous -125 mmHg. Interval dressing changes were performed every 48-72 hours. Overall improvement in wound size and disposition was noted over the next month of care (Figure 9). Leg parasthesias improved gradually and completely resolved within the first postoperative month. Limb pain resolved within this time period as well.

**Figure 10:**
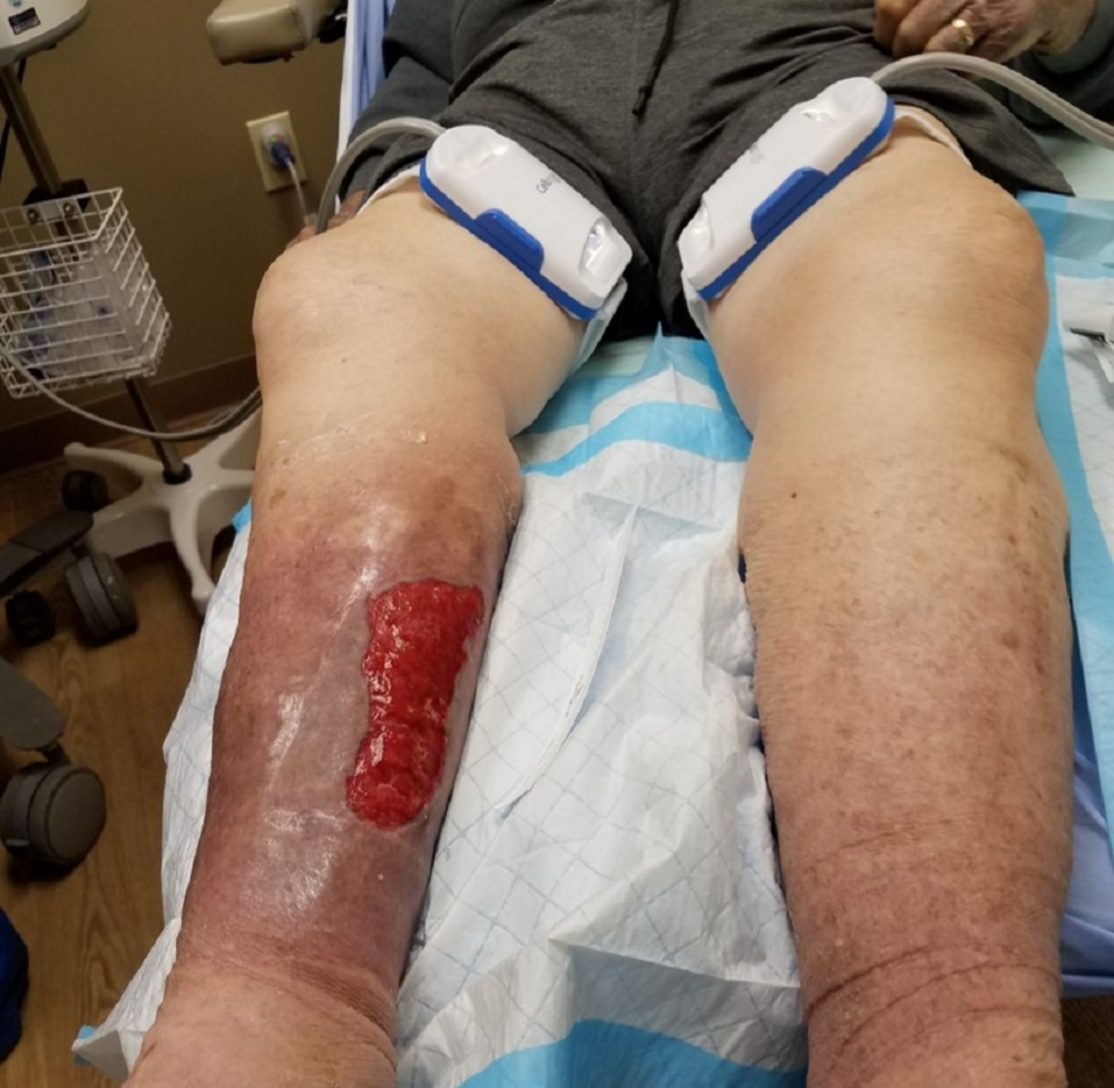
Double Cellutome™ harvester technique.

Approximately six weeks from surgery, the wound bed was noted to be entirely granular with no depth. NPWT was discontinued and the patient was offered a traditional split-thickness skin graft or automated suction blister epidermal harvesting and grafting using the Cellutome™ device. Risks and benefits of both techniques were discussed with the patient. He opted for the minimally invasive automated technique. The procedure was done in the clinic setting and no anesthesia was required. Because of the wound size, bilateral harvesters were utilized to procure the epidermis from both the right and left thighs (Figure 10).

**Figure 11:**
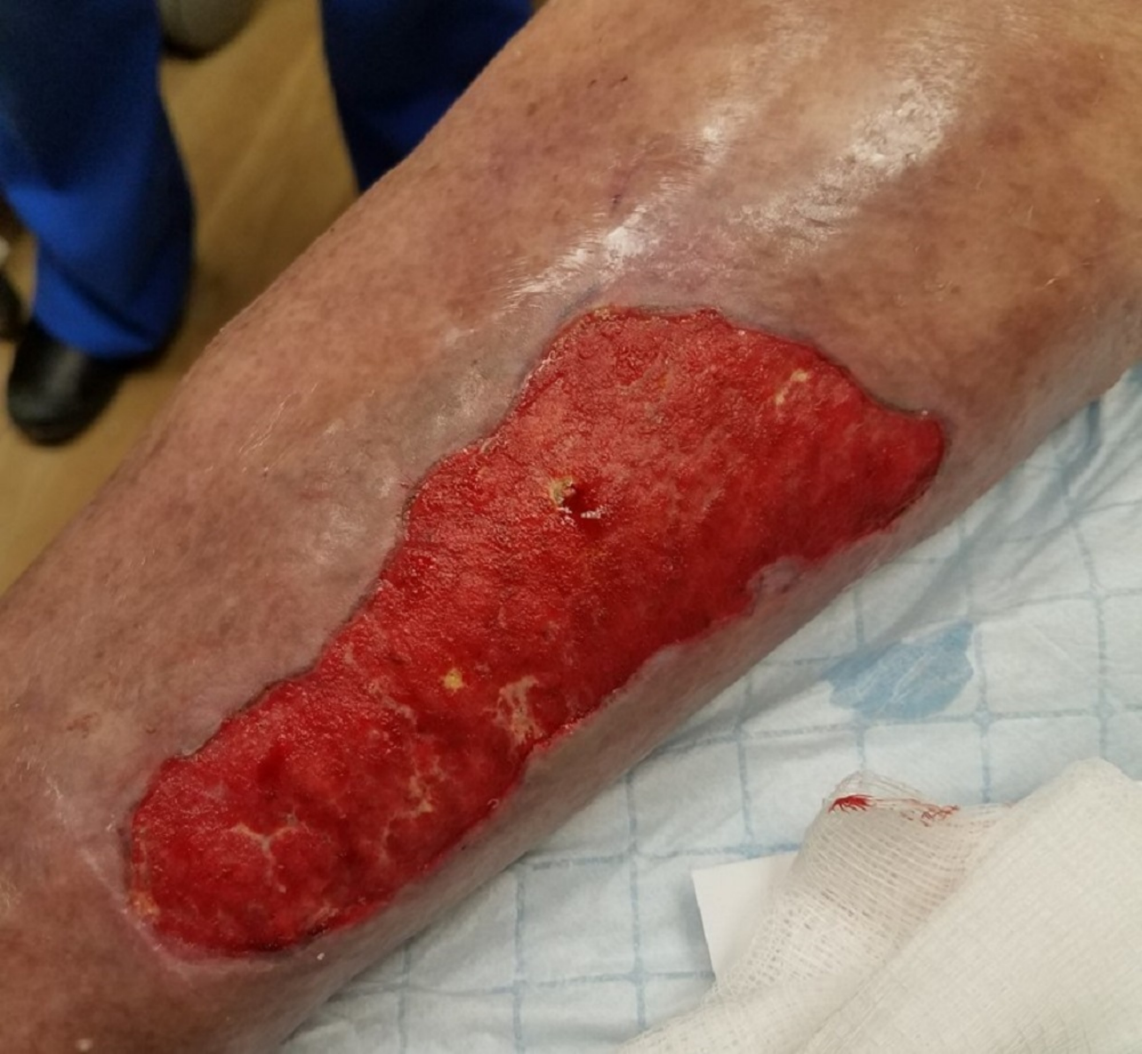
Two weeks status post initial suction blister grafting using the Cellutome™ device (KCI, an Acelity company, San Antonio, TX, USA).

**Figure 12:**
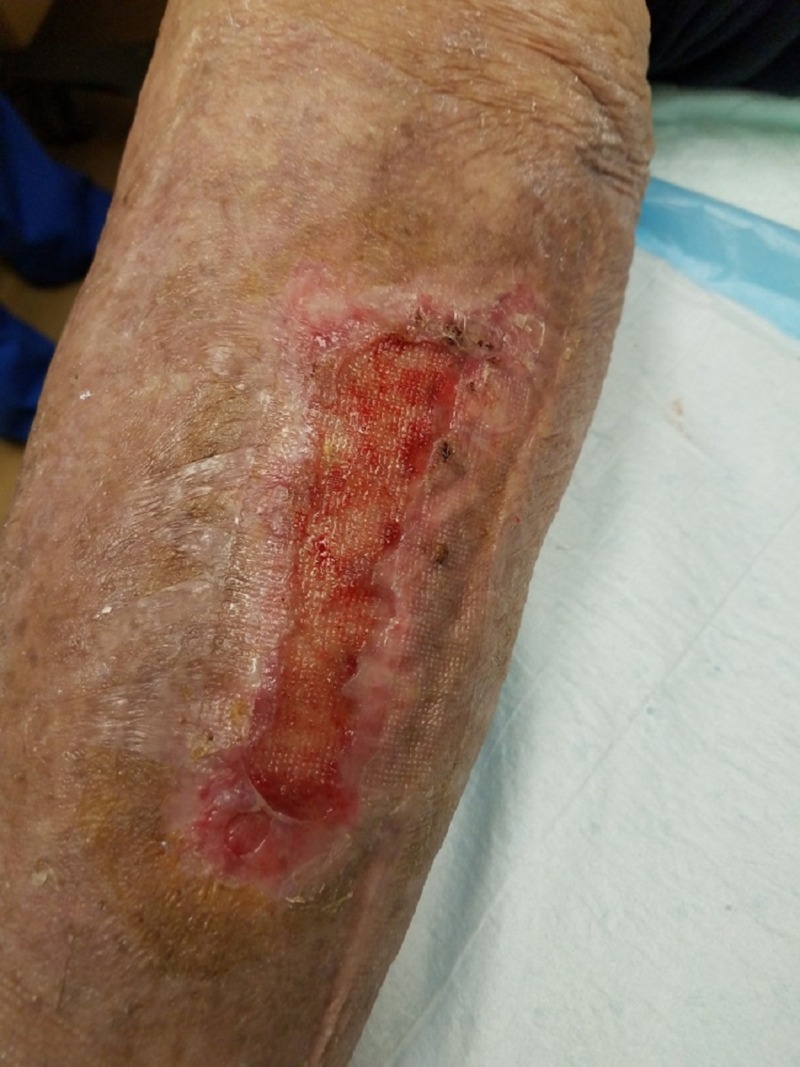
Wound approximately one month after second epidermal harvesting and grafting technique.

Postoperative care included simple bandage protection of the donor and recipient sites together with edema control in the form of disposable compression bandages (Figure 11). Normal daily activities and physical therapy were continued. Automated epidermal suction blister harvesting and grafting was carried out in serial fashion in the light of the wound size and was performed a total of three times with procedures spaced six to eight weeks apart (Figure 12).

**Figure 13:**
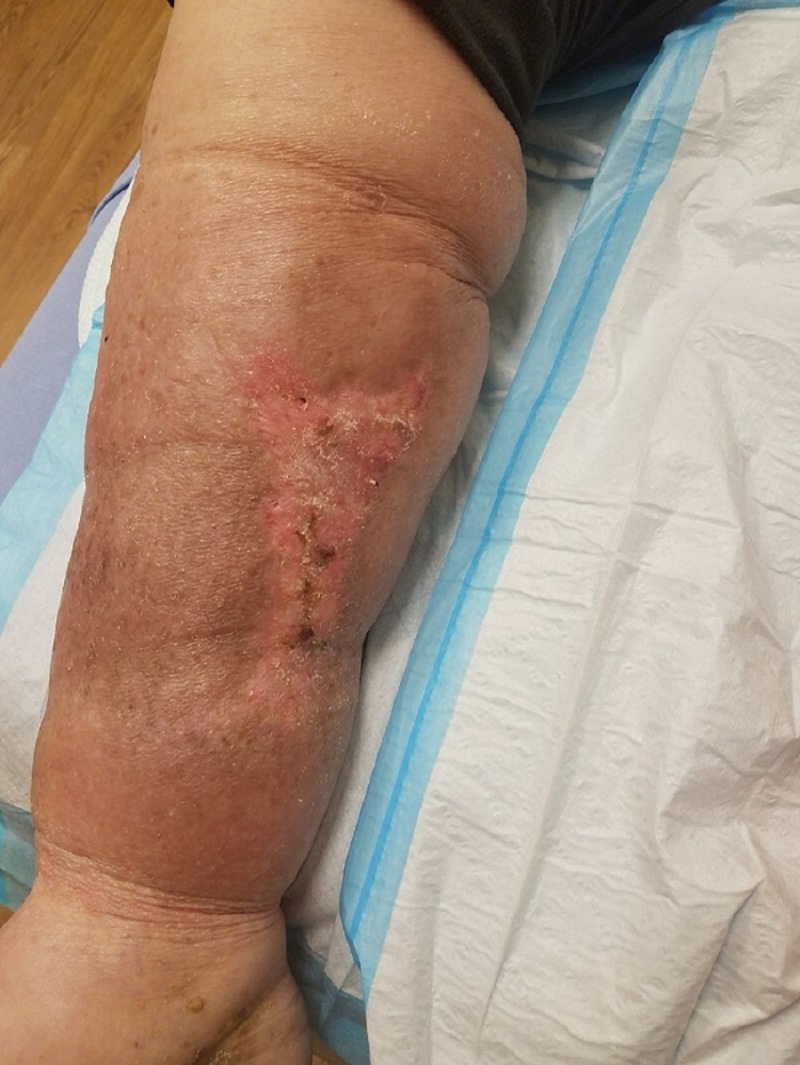
Complete closure achieved at six months.

**Figure 14:**
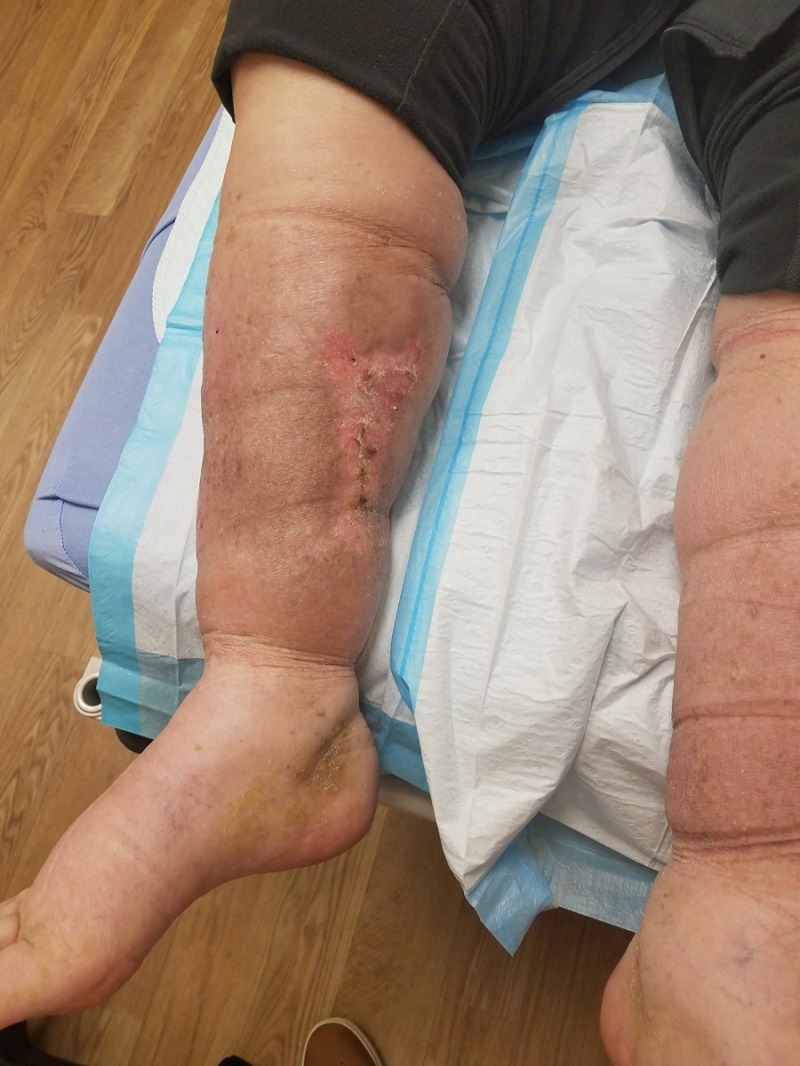
Complete closure and return to normal function at six months.

Complete closure was achieved approximately six weeks after the third epidermal harvesting and grafting technique (Figures 13-14). The patient recovered completely without any loss of sensation or function.

## Discussion

Injury and wound complications from acute extremity compartment syndrome can range in severity. The classic "Five Ps" of compartment syndrome (pain, pallor, paresthesia, pulselessness, and paralysis) need not all be present to make the diagnosis. They may be present in their entirety in more severe cases, or partially in varying degrees. Compartment syndrome develops from either intracompartmental swelling or external compression of an extremity. This particular case was thought to represent severe soft tissue sequela from acute compartment syndrome in which the patient presented much later after the initial traumatic event. Because of the patient’s history, clinical presentation, and extensive soft tissue involvement, the diagnosis of compartment syndrome sequela was favored over hematoma alone. The etiology was from significant, sustained, external lower extremity compression trauma to the calf. The anatomical compartment most significantly affected was the posterior superficial compartment. Surgical management included extensive debridement, hematoma evacuation, exploration of neurovascular structures, and pulsed lavage. Because of the significant wound size, tissue void, amount of tissue necrosis, and exudate, NPWTi-d was initiated and favored over NPWT alone. Decreased healing time is certainly a goal in wound care, and studies of NPWTi-d have demonstrated the development of more granulation tissue in a shorter time compared to NPWT alone. Specifically, Lessing et al. showed that wounds treated with NPWTi-d had 43% more granulation tissue present after seven days compared to NPWT alone [19]. In the place of saline, the author has occasionally used anti-infective reagents such as diluted hypochlorous acid or Prontosan® as the irrigant in NPWTi-d. However, in this specific clinical practice, solutions other than normal saline have been reserved for cases in which infection or extensive bioburden is thought to be present in addition to necrosis alone. As stated previously in this article, NPWTi-d is not a therapy for the treatment of wound infection or a mechanism to deliver intravenous antibiotics. It can, however, facilitate wound healing by cleansing the wound through the instillation of topical antiseptic/antimicrobial wound cleansers that can help manage bacterial bioburden. In choosing the primary dressing to use for NPWTi-d in this case, a novel dressing consisting of polyurethane reticulated open cell foam with through holes (V.A.C. VeraFlo Cleanse Choice™ Dressing) was selected. This particular dressing was chosen because its design is thought to aid in the cleansing of larger wounds with thick and copious exudate. With respect to solution amount, dwell time, and NPWT time after dwell, in this case, and other cases using NPWTi-d, the author has used similar parameters found in the literature [20]. For example, Kim et al. have demonstrated positive outcomes with NPWTi-d when a dwell time of six minutes was used followed by three-and-a-half hours of continuous NPWT [15]. Once the wound was ready for skin grafting, automated suction blister epidermal harvesting and grafting using the Cellutome™ device was carried out. The patient chose this technique because of the advantages over split-thickness skin grafting in some cases. These include essentially no donor site morbidity, minimal patient discomfort, and the ability to harvest tissue without surgery or anesthesia. Automated epidermal suction blister harvesting and grafting was carried out in serial fashion in the light of the wound size and was performed a total of three times with procedures spaced six to eight weeks apart. Complete wound closure was seen in six months. Ambulatory status and return to normal limb function were achieved much sooner, in approximately two months. During his treatment course, the patient underwent only one major operation with a hospital stay of six days for NPWTi-d. The majority of his care was provided in an outpatient wound care clinic and nursing home. Ultimately he returned to an assisted living environment where he resided at initial presentation.

## Conclusions

Acute compartment syndrome sequela of the lower leg can be treated successfully after surgery with negative pressure wound therapy with instillation and dwell time utilizing a novel dressing (V.A.C. VeraFlo Cleanse Choice™ Dressing) and serial automated suction blister epidermal harvesting and grafting with the Cellutome™ device. The outcome for this patient was very favorable, resulting in complete wound healing, limb preservation, return to normal function, and daily activities.
